# Serum from human burn victims impairs myogenesis and protein synthesis in primary myoblasts

**DOI:** 10.3389/fphys.2015.00184

**Published:** 2015-06-16

**Authors:** Katie L. Corrick, Michael J. Stec, Edward K. Merritt, Samuel T. Windham, Steven J. Thomas, James M. Cross, Marcas M. Bamman

**Affiliations:** ^1^Department of Cell, Developmental, and Integrative Biology, University of Alabama at BirminghamBirmingham, AL, USA; ^2^UAB Center for Exercise Medicine, University of Alabama at BirminghamBirmingham, AL, USA; ^3^Department of Surgery, University of Alabama at BirminghamBirmingham, AL, USA; ^4^Department of Surgery, University of Texas Health Science CenterHouston, TX, USA; ^5^Geriatric Research, Education, and Clinical Center, Birmingham VA Medical CenterBirmingham, AL, USA

**Keywords:** burn, inflammation, myogenesis, myoblast, myotube, muscle protein synthesis

## Abstract

The pathophysiological response to a severe burn injury involves a robust increase in circulating inflammatory/endocrine factors and a hypermetabolic state, both of which contribute to prolonged skeletal muscle atrophy. In order to characterize the role of circulating factors in muscle atrophy following a burn injury, human skeletal muscle satellite cells were grown in culture and differentiated to myoblasts/myotubes in media containing serum from burn patients or healthy, age, and sex-matched controls. While incubation in burn serum did not affect NFκB signaling, cells incubated in burn serum displayed a transient increase in STAT3 phosphorlyation (Tyr705) after 48 h of treatment with burn serum (≈ + 70%; *P* < 0.01), with these levels returning to normal by 96 h. Muscle cells differentiated in burn serum displayed reduced myogenic fusion signaling (phospho-STAT6 (Tyr641), ≈−75%; ADAM12, ≈-20%; both *P* < 0.01), and reduced levels of myogenin (≈−75%; *P* < 0.05). Concomitantly, myotubes differentiated in burn serum demonstrated impaired myogenesis (assessed by number of nuclei/myotube). Incubation in burn serum for 96 h did not increase proteolytic signaling (assessed via caspase-3 and ubiquitin levels), but reduced anabolic signaling [p-p70S6k (Ser421/Thr424), −30%; p-rpS6 (Ser240/244), ≈-50%] and impaired protein synthesis (−24%) (*P* < 0.05). This resulted in a loss of total protein content (−18%) and reduced cell size (−33%) (*P* < 0.05). Overall, incubation of human muscle cells in serum from burn patients results in impaired myogenesis and reduced myotube size, indicating that circulating factors may play a significant role in muscle loss and impaired muscle recovery following burn injury.

## Introduction

Severe burn injury affecting greater than 20% of the total body surface area (TBSA) causes a robust inflammatory response and an increased metabolic demand which contribute to rapid skeletal muscle catabolism and ultimately lead to long-term muscle wasting (reviewed in Wolfe, 1981; Pereira et al., 2005; Porter et al., 2013). The progressive loss of muscle mass negatively impacts the rate of recovery by delaying wound healing, increasing the risk of infection, prolonging mechanical ventilator requirements, and delaying mobilization (reviewed in Chang et al., 1998). Loss of 25% of total body nitrogen can be fatal, and without nutritional supplementation beyond normal caloric requirements, this amount of protein loss can be reached within 3–4 weeks after the burn injury (Pereira et al., 2005). While a combination of nutritional supplementation and administration of anabolic hormones may attenuate muscle loss in burn patients, a greater understanding of the cellular mechanisms regulating muscle catabolism in this population could help lead to the development of more targeted, effective therapies.

Depending on the severity of the burn, the hypermetabolic state can last up to several years post-injury (Jeschke et al., 2007, 2011) and is a likely contributor to the difficulty of returning muscle mass to pre-injury levels. The hypermetabolic state in burn patients is characterized by increased body temperature, resting energy expenditure, glycogenolysis, proteolysis, lipolysis, and futile substrate cycling (Wilmore and Aulick, 1978; Herndon and Tompkins, 2004), which can contribute to significant muscle protein degradation. Simultaneously, these patients are under the burden of a hyperinflammatory state, characterized by a substantial rise in pro-inflammatory cytokines [e.g., interleukin (IL)-6, tumor necrosis factor (TNF)-α], many of which have negative effects on muscle regeneration/growth (Jeschke et al., 2008; Merritt et al., 2012). It has been clear for some time that burn injury exerts profound effects on skeletal muscle at sites distant from the actual injury, indicating humoral factors such as inflammatory cytokines and stress hormones are at least partially responsible. Recently, our lab has shown significantly elevated calcium-mediated proteolytic signaling and ubiquitin-proteasome activity in skeletal muscle of human burn patients distant from the burn (Merritt et al., 2012), indicating hyperactive muscle catabolism. Concomitantly, these patients had robust elevations in circulating cytokines (up to 70-fold greater than control), as well as elevated inflammatory muscle gene expression (Merritt et al., 2012, 2013b), suggesting that systemic factors may indeed play a key role in muscle wasting in burn patients.

Whole organism burn models make it difficult to discern whether muscle protein loss is due primarily to intrinsic (e.g., hypermetabolism causing muscle proteolysis) or systemic (e.g., increases in circulating cytokines and/or endocrine factors) effects of the burn injury. Therefore, the aim of this project was to use an *in vitro* system to determine the effect of serum from severely burned patients (≈30% TBSA burn) on the growth, differentiation, and cellular signaling of skeletal muscle cells obtained from healthy humans. We hypothesized that treatment with burn serum would impair growth and differentiation of human myotubes, and reduce rates of protein synthesis, which would provide *in vitro* evidence to support the central hypothesis that circulating factors exert profound effects on non-burned skeletal muscle of burn patients.

## Materials and methods

### Subjects

Six patients with 20–60% TBSA burns were recruited from the regional burn center. Blood samples from these individuals were obtained within 3–10 days following burn injury, and serum was isolated by centrifugation. Serum from sex and age matched healthy adults (*n* = 6) was obtained from the Core Muscle Research Laboratory's de-identified serum bank and used as control for all experiments. The study was approved by the local Institutional Review Board and patients gave written informed consent prior to participation.

### Cell culture

Satellite cells isolated from the vastus lateralis of three healthy males (≈30 year) were obtained from the Core Muscle Research Laboratory's de-identified cryostorage bank. Serum from the six burn-injured patients was pooled and designated as “burn” serum. Serum from healthy age and sex matched donors was pooled and designated as “control” serum. Satellite cells were grown to ≈80% confluence, and then treated with differentiation media (DMEM containing 100 μL/mL streptomycin, 100 U/mL penicillin, and 5% human serum) with either 5% burn or control serum in side-by-side parallel experiments. Differentiation media was changed every 48 h, and cells were lysed at 48 and 96 h. Protein synthesis rates were assessed at 96 h using the surface sensing of translation (SUnSET) technique (Schmidt et al., 2009), which involved a 30 min puromycin pulse (1 μM; Sigma-Aldrich, St. Louis, MO) prior to lysing.

### Immunocytochemistry

To examine the effects of burn serum on myogenic differentiation and myotube size, cells differentiated for 96 h were stained for myosin heavy chain protein expression. After being fixed with methanol for 20 min and washed with PBS, cells were blocked in 5% goat serum with 0.1% Triton X-100 for 20 min. Cells were incubated overnight at 4°C with MF-20 primary antibody (Developmental Studies Hybridoma Bank; 25 μg/mL) in 5% goat serum. After washing in PBS, cells were incubated in secondary Ab (Alexa Fluor 488 goat-anti-mouse, Life Technologies, Grand Island NY; 1:200) in 1% goat serum at room temperature for 1 h. Cells were washed with PBS and coverslips were mounted with ProLong Gold (Life Technologies) with DAPI (4′,6-diamidino-2-phenylindole) for nuclei labeling. Using an Olympus BX51 fluorescent microscope with an Olympus MagnaFire SP camera (S99810), images were captured at 10X and 4X magnifications for analysis of myotube fusion and volume, respectively. Image analysis of myotube fusion (i.e., the number of nuclei per myotube) was performed using Image-Pro Plus 6.0 software. The percentage of myotubes containing five or more nuclei was used for analysis as a marker of myogenic fusion. Analysis of myotube volume was performed according to previously established protocols (Agley et al., 2012) using Adobe Photoshop version CS6.

### Immunoblotting

**Figure 1:**
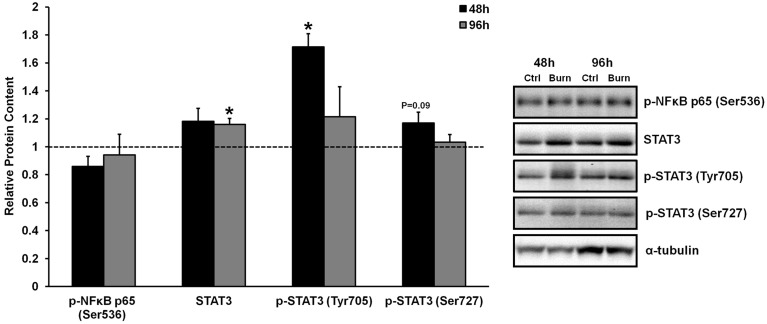
**Effects of burn serum on inflammatory NF κB and STAT3 signaling**. ^*^Different from control at respective time point; *P* < 0.05. Dotted line represents control values. Representative blots for α-tubulin demonstrate equal loading between burn and control lanes.

Cells were lysed in 150 μL of ice cold RIPA buffer containing protease and phosphatase inhibitors. Cell lysate was centrifuged at 5000 g for 5 min at 4°C. The supernatant was assayed for protein content using the bicinchonic acid technique with BSA as a standard. Cell protein lysate (20 μg) was resolved on 4–12% SDS-PAGE gels and transferred to PVDF membranes. Analysis of inflammatory, fusion/differentiation, and anabolic/catabolic signaling pathways was performed by standard immunoblotting. Antibodies raised against phosphorylated (Ser536) and total NFκB p65, phosphorylated (Ser421/Thr424) and total p70S6k, phosphorylated (Ser240/244) and total rpS6, phosphorylated (Tyr705 and Ser727) and total STAT3, phosphorylated (Tyr641) and total STAT6, ADAM12, ubiquitin, caspase-3, and α-tubulin were from Cell Signaling Technologies (Danvers, MA), while antibodies for myogenin and MyoD were from Santa Cruz Biotechnology Inc. (Santa Cruz, CA) and anti-puromycin antibody was from Millipore (Billerica, MA). Primary antibodies were diluted 1:1000 in PBST and 5% goat serum (monoclonal antibodies) or 2% milk/2% BSA (polyclonal antibodies). HRP-conjugated secondary antibody (Pierce Thermoscientific, Rockford, IL) was used at 1:50,000 (w/v) followed by chemiluminescent detection in a BioRad (Hercules, CA) ChemiDoc imaging system with band densitometry performed using BioRad Quantity One software (software package 4.5.1). Equal loading was verified by Ponceau-S staining of the PVDF membranes prior to immunoblotting. In addition, α-tubulin served as a loading control within each time point (i.e., 48 or 96 h). There were no statistically significant differences in α-tubulin levels between control vs. burn-treated cells in any of the control vs. burn comparisons at 48 or 96 h. While not a primary comparison, we also found no statistically significant differences in α-tubulin levels between 48 and 96 h; however, the appearance of an increase (e.g., Figure 1) is not unexpected. During myogenesis, microtubule accumulation and reorganization occurs, which could lead to an accumulation of α-tubulin from 48 to 96 h of differentiation. It is therefore not surprising that others have noted an increased amount of total tubulin content during myoblast differentiation (Gundersen et al., 1989). Regardless, all of our comparisons between burn and control occurred at single time points (48 or 96 h), not across time.

### Statistics

For each experiment, cells treated with burn serum were studied in parallel with cells treated with healthy, control serum. All data in each burn serum experiment were then normalized to control serum conditions. Unpaired *t*-tests were used to determine differences in myogenic fusion, myotube volume, and signaling protein levels between cells treated with burn and control serum. Significance was accepted at *P* < 0.05.

## Results

### Effects of burn serum on inflammatory signaling

Inflammatory cytokine signaling through the well-characterized TNF-α/NFκB and IL-6/STAT3 pathways has been shown to induce skeletal muscle atrophy *in vivo* (Coletti et al., 2005; Haddad et al., 2005). We have previously shown that serum from burned patients contained 5- and 70-fold higher levels of TNF-α and IL-6 compared to control serum, respectively, although heightened TNF- α/NFκB or IL-6/STAT3 signaling were not evident in the muscles of these patients (Merritt et al., 2012). Similarly, treatment of primary human muscle cells with burn serum in the current study did not induce robust TNF- α/NFκB signaling. Specifically, treatment with burn serum did not increase the phosphorylation of NFκB p65 at Ser536 (which directs NFκB nuclear translocation and activation) at any time when compared to control serum (Figure 1). There was a significant increase in STAT3 phosphorlyation at Tyr705 after 48 h of treatment with burn serum (+71%; *P* < 0.01), but these levels returned to normal by 96 h. Increased phosphorylation at the site which directs STAT3 transcriptional activity (Ser727) was not found (*P* = 0.09 at 48 h). By 96 h, there was a slight increase in total STAT3 protein content (+15%; *P* < 0.001) in cells treated with burn serum. These data indicate that serum obtained from burn patients does not induce NFκB signaling in muscle cells, and induces a prolonged activation of IL-6/STAT3 signaling.

### Effects of burn serum on myogenic signaling

**Figure 2:**
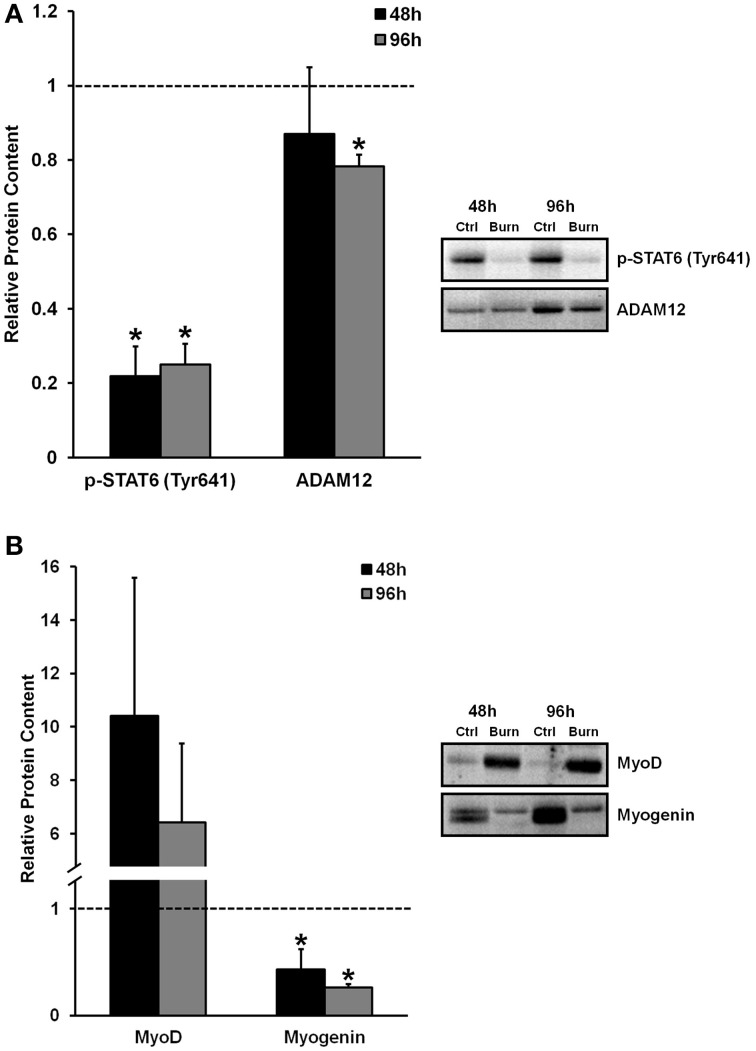
**Effects of burn serum on markers of myogenic fusion (A) and myogenic differentiation (B)**. ^*^Different from control at respective time point; *P* < 0.05. Dotted line represents control values.

The activation, proliferation, and differentiation of muscle satellite cells are required for muscle regeneration from injury, and fusion of differentiated/committed satellite cells (i.e., fusion-competent myoblasts) with existing myofibers supports myofiber hypertrophy. We found reduced levels of proteins that regulate myogenic fusion (Figure 2A) and differentiation (Figure 2B) in muscle cells differentiated in burn serum, confirming that burn serum creates an anti-myogenic environment, which likely contributes to impaired muscle regrowth following burn injury-induced muscle atrophy. Levels of p-STAT6 (Tyr641), a marker of IL-4 mediated myoblast fusion, were ≈75% lower at 48 and 96 h (*P* < 0.01 for both) in muscle cells incubated in burn serum compared to control. Additionally, levels of ADAM12 (disintegrin and metalloproteinase domain-containing protein 12), a transmembrane metalloproteinase that is required for myoblast fusion (Galliano et al., 2000), was 22% lower (*P* < 0.01) after 96 h of differentiation in burn serum.

Regarding myogenic differentiation, myogenin, the putative myogenic regulatory factor responsible for terminal differentiation, increased as expected from 48 to 96 h of differentiation in control, but was substantially suppressed in response to burn serum at both 48 and 96 h compared to control (57 and 74% lower, respectively; *P* < 0.05). MyoD, an early marker of commitment to the myogenic lineage, appeared to be expressed at extremely high levels with burn serum treatment (10-fold higher at 48 h, and six-fold higher at 96 h, albeit non-significant due to high variability). Together these findings suggest burn serum suspended myoblasts in an early phase of differentiation; thereby limiting terminal differentiation and fusion.

### Effects of burn serum on protein metabolism signaling

**Figure 3:**
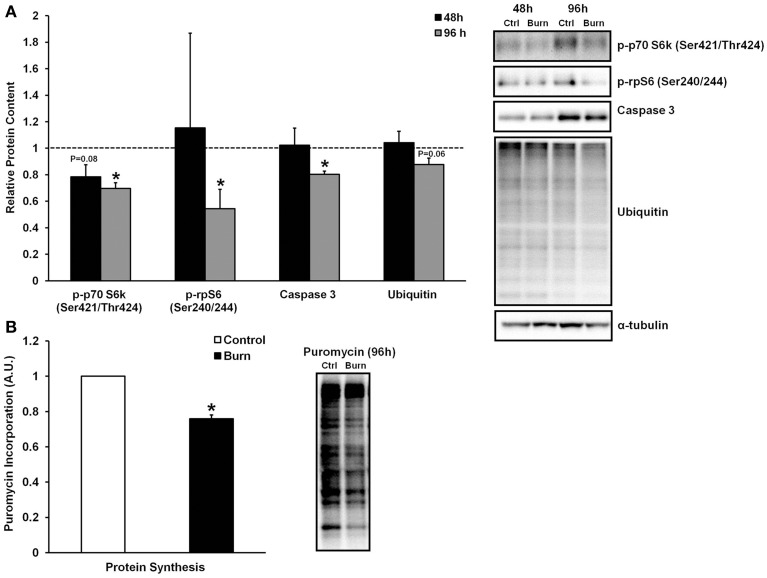
**Effects of burn serum on anabolic/catabolic signaling (A) and protein synthesis measured via the SUnSET method (B)**. ^*^Different from control at respective time point; *P* < 0.05. Dotted line represents control values.

Skeletal muscle loss caused by burn injury is due to an increase in muscle protein breakdown, a decrease in muscle protein synthesis, or a combination of both of these factors. We therefore examined both markers of protein breakdown and synthesis in myotubes treated with burn and control serum (data shown in Figure 3). Interestingly, caspase-3 levels were significantly lower in muscle cells treated with burn serum for 96 h (−20%; *P* < 0.01), and total protein ubiquitination tended to be lower as well (−12%; *P* = 0.06) when compared to control. Thus, burn serum did not appear to up-regulate proteolysis in this *in vitro* model. On the other hand, anabolic signaling was suppressed in muscle cells treated with burn serum for 96 h, as evidenced by significant decreases in p-p70S6k (Ser421/Thr424) (−30%) and p-rpS6 (Ser240/244) (−46%) (both *P* < 0.05). In parallel with these findings, protein synthesis rate, measured via the SUnSET method, was suppressed 24% (*P* < 0.001) in muscle cells treated with burn serum compared to control (Figure 3B). These *in vitro* findings suggest serum from burn patients causes negative protein balance by blunted anabolic signaling and protein synthesis but not up-regulated proteolysis.

### Effects of burn serum on myoblast fusion and myotube size

**Figure 4:**
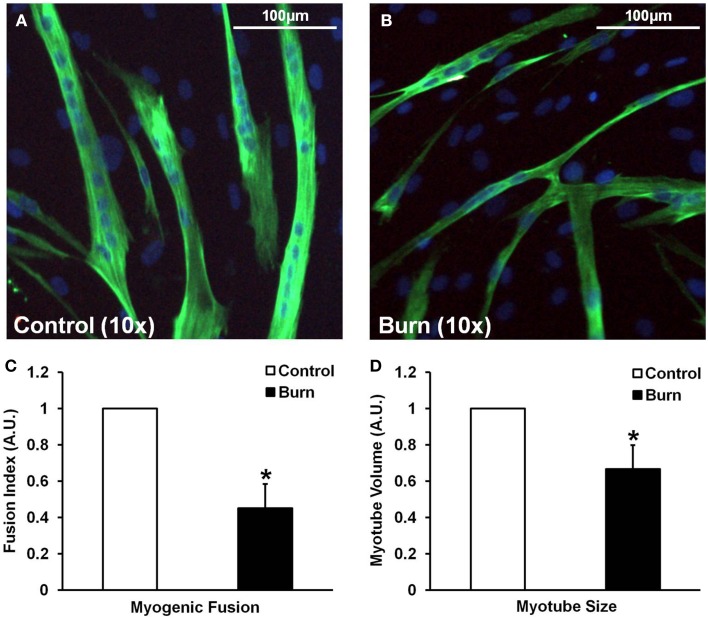
**Effects of burn serum on myogenic fusion (C) and myotube size (D). (A,B)** are representative immunocytochemical images of myotubes differentiated in control **(A)** or burn **(B)** serum. ^*^Different from control at respective time point; *P* < 0.05.

In agreement with the signaling data, the addition of burn serum to healthy muscle cells ultimately resulted in reduced myotube formation and myotube size (representative images shown in Figures 4A,B). Incubation in burn serum for 96 h did not result in cell death (assessed by total nuclei/image; control: 248 ± 53 nuclei/image; burn: 237 ± 38 nuclei/image), but did reduce the number of nuclei per myotube (55% fewer myotubes with 5+ nuclei; *P* < 0.05), indicating impaired fusion compared to control (Figure 4C). Additionally, myotubes grown in burn serum had significantly reduced total protein content (−18%, *P* < 0.05; data not shown) and myotube size was 33% smaller (*P* < 0.05) compared to control (Figure 4D).

## Discussion

Although the effects of severe burn injury on skeletal muscle loss are well-documented (Wolfe, 1981; Hart et al., 2000; Pereira et al., 2005; Jeschke et al., 2008, 2011), the major mechanisms driving net negative muscle protein balance in burn patients are largely unknown. Changes in whole body metabolism as well as changes in the systemic milieu (e.g., circulating cytokines, endocrine factors) both likely contribute to muscle wasting. Here, using an *in vitro* system, we show that serum obtained from burn patients significantly reduces myoblast differentiation and fusion, and myotube size in healthy primary human muscle cells. The effect of burn serum on reducing myoblast differentiation/fusion seems to be regulated via decreases in signaling pathways directing fusion (e.g., p-STAT6 and ADAM12) as well as dysregulation of myogenic regulatory factors (e.g., myogenin and MyoD). Interestingly, the effect of burn serum on reducing myotube size does not appear to be driven by an upregulation of protein breakdown; rather, a decrease in anabolic signaling (e.g., p70S6k/rpS6) and cellular protein synthesis. Overall, these results indicate that burn serum, on its own, can cause significant decreases in muscle cell size via impaired fusion and suppression of cellular protein synthesis.

Following a severe burn injury, the body presents a stress response, releasing abundant amounts of pro-inflammatory cytokines, and hormones (Jeschke et al., 2008). Marked increases in circulating cytokines would be expected to affect skeletal muscle in a negative manner, as excess levels of pro-inflammatory cytokines such as TNF-α and IL-6 can activate downstream NFκB and STAT3 signaling, respectively, ultimately leading to muscle wasting and impaired muscle regeneration (Szalay et al., 1997; Coletti et al., 2005; Haddad et al., 2005; Merritt et al., 2013a). We (Merritt et al., 2012) and others (Jeschke et al., 2008) have previously reported robust elevations of these cytokines (e.g., >100-fold elevations in IL-6) in serum obtained from burn patients, although we have shown that, despite these elevations, pro-inflammatory NFκB and STAT3 signaling in non-burned skeletal muscle tissue is not elevated (Merritt et al., 2012). The results of the present study corroborate with these findings, as healthy muscle cells treated with burn serum *in vitro* did not demonstrate an increase in NFκB signaling. While there was a significant increase in p-STAT3 (Tyr705) in response to 48 h burn serum treatment, this effect was transient, with levels of p-STAT3 (Tyr705) returning to normal by 96 h. It is important to note that phosphorylation of STAT3 at Tyr705 is required for STAT3 dimerization, nuclear translocation, and DNA binding, however, phosphorylation of STAT3 at Ser272 is required for maximal transcriptional activity of STAT3 dimers in the nucleus (Wen et al., 1995). Phosphorylation of STAT3 on Ser727 only displayed a trend toward an increase following 48 h of burn serum treatment (+17%, *P* = 0.09), and normal levels by 96 h. Taken together, the increase in p-STAT3 (Tyr705) at 48 h, and the mild, non-significant increase in p-STAT3 (Ser727) at 96 h indicate that burn serum does not induce a prolonged inflammatory response in muscle cells via STAT3. Rather, robust activation of STAT3 (and perhaps even NFκB) signaling are likely to occur at earlier time points following burn serum treatment *in vitro*, as well as during early time points following acute burn injury *in vivo*.

Recovery from a burn injury is likely to involve the activation and differentiation of skeletal muscle satellite cells, as these cells would be necessary for muscle tissue repair if any damage has occurred during the initial injury. Additionally, myonuclear addition via fusion of satellite cells into existing myofibers may help facilitate protein synthesis within the myofiber during long-term recovery from the injury (Adams and Bamman, 2012). Wu et al. (2013) have recently shown that satellite cells are activated in rat muscle following burn injury *in vivo*, and that burn serum from rats can activate myoblasts *in vitro*, although they did not assess the effects of burn serum on the differentiation of these cells. Here, we show that treatment with burn serum reduces the myogenic fusion and differentiation of healthy, human muscle cells *in vitro*. Myoblast fusion occurs in two phases: in the first phase, myoblasts fuse together to form a nascent myotube, and in the second phase, myoblasts fuse with nascent myotubes to form mature myotubes (Horsley and Pavlath, 2004). Thus, we chose to measure signaling proteins that regulate both the first (ADAM12) and second (IL-4/STAT6) phases of myoblast fusion. Burn serum treatment reduced protein levels of ADAM12, a transmembrane metalloprotease that is required for phase 1 myoblast fusion (Galliano et al., 2000), and the phosphorylation state of STAT6 (Tyr641), which is activated via IL-4 and directs phase 2 fusion (Horsley et al., 2003). These results show that burn serum exerts profound effects on reducing both phase 1 and phase 2 fusion in myogenic cells. Relevant to these data, muscle cells differentiated in burn serum had significantly reduced expression of myogenin (less than half that of control), and robustly elevated levels of MyoD (6-10-fold higher) compared to control, indicating aberrant regulation of the myogenic program caused by burn serum. Altogether, dysregulation of these pathways regulating terminal differentiation and myogenic fusion resulted in a quantifiable reduction in the number of myonuclei per myotube. Clearly, burn serum can reduce the differentiation and fusion of primary human myoblasts, which likely translates into impaired muscle recovery following a burn injury.

The most important determinant of how much skeletal muscle is lost following a burn injury is the net protein balance of the patient. Hart et al. (2000) have shown that a negative net protein balance persists up to 9 months following severe burn injury. This indicates that, in burn patients, muscle protein breakdown is increased, and/or protein synthesis is decreased. Muscle protein breakdown is due to hypermetabolism, with preferential breakdown of skeletal muscle as a primary source of nitrogen and energy (Pereira et al., 2005). In accordance with this, we have previously shown an increased expression of the muscle-specific E3 ubiquitin ligases, atrogin-1 and MuRF1, as well as markers of proteolytic signaling (e.g., ubiquitin-proteasome activity and calcium-mediated proteolysis) in human whole muscle tissue following burn injury (Merritt et al., 2012, 2013b). The results of the current study do not agree with our previous findings, as levels of caspase-3 were actually lower in muscle cells treated with burn serum, and total ubiquitination tended to be lower (albeit non-significant) when compared to control. Levels of caspase-3 could be indicative of muscle protein breakdown, as caspase-3 cleaves myofibrils into monomeric actin and myosin prior to degradation via the ubiquitin-proteasome system (Du et al., 2004). Interestingly, however, caspase-3 is also required for muscle differentiation, and inhibition of caspase-3 severely reduces myogenesis (Fernando et al., 2002). In the current study, levels of caspase-3 increased during the time course of differentiation, with the control serum-treated group increasing caspase-3 levels ≈50% from 48 to 96 h, while the burn-treated group only increased caspase-3 levels ≈20% from 48 to 96 h (data not shown). Thus, based on these observations, the reduced caspase-3 content in the burn-serum treated group may not be a reliable marker of overall protein breakdown; rather, it may just be a consequence of impaired myogenesis in this group. Nevertheless, there was not a significant increase in total protein ubiquitination following treatment with burn serum, suggesting burn serum may not play a major role in the muscle proteolysis that has been observed in whole tissue studies.

While we did not find any increases in markers of protein breakdown, the significant reduction in myotube size following treatment with burn serum was linked to an attenuation of protein synthesis. Indeed, we found that protein synthesis was reduced by ≈25% in myotubes grown in burn serum vs. control. In order to elucidate the mechanisms responsible for repressed anabolism in burn serum treatment, we examined the putative mammalian target of rapamycin (mTOR) pathway, which is a major pathway involved in muscle mass regulation. mTOR is a protein kinase that integrates a variety of intracellular signals, including those arising due to nutrient availability (or lack thereof), growth factor stimulation, and stress responses (Frost and Lang, 2011; Laplante and Sabatini, 2012). As part of the active mTORC1 complex, mTOR phosphorylates several downstream targets, including the 70-kilodalton S6 protein kinase (p70S6k), which, in turn, phosphorylates ribosomal protein S6 (rpS6). Treatment of healthy muscle cells with burn serum effectively reduced phosphorylation of the auto-inhibitory domain (Ser421/Thr424) of p70S6k by ≈30%, and in turn, reduced levels of p-rpS6 (Ser240/244) by ≈30%. Reduced p70S6k/rpS6 signaling is likely a major factor contributing to attenuated protein synthesis in myotubes grown in burn serum; however, the mechanisms regulating this are not known.

Although we have shown definitive evidence that serum obtained from burn patients is a sufficient stimulus to reduce myogenesis and blunt protein synthesis, it remains unknown exactly which factors in the serum contribute to these effects. Numerous immunologic and endocrine factors have been found to be drastically different in serum obtained from burn patients when compared to control serum, such as: complement, C-reactive protein, interferon γ, TNF-α, interleukins-1β, 2, 4, 5, 6, 7, 8, 10, 12, 13, and 17, catecholamines, insulin-like growth factor-1 (IGF-1), IGF-1 binding protein-3 (IGFBP-3), growth hormone (GH), testosterone, thyroxine (T4), and cortisol (Crum et al., 1990; Jeffries and Vance, 1992; Jeschke et al., 2008; Merritt et al., 2012). Our data suggest that, despite large increases in circulating TNF-α and IL-6, the rise in these pro-inflammatory cytokines is not enough to activate downstream NFκB and STAT3 signaling in skeletal muscle for a prolonged period of time. However, it is important to note that the burn serum in this study comprised only 5% of the total media in these experiments; thus, the muscle cells may have had a significant sustained inflammatory signaling response if they were exposed to a higher concentration of serum. Knowledge from previous studies (Moller et al., 1991; Jeschke et al., 2008) showing that IGF-1 and IGFBP-3 are reduced in the serum of burn patients makes it interesting to speculate that the reduction in p70S6k signaling was due to reduced availability of IGF-1 to bind to its cognate receptor and activate the putative IGF-1/Akt/mTOR/p70S6k pathway. Rescue of this signaling pathway via administration of IGF-1 or IGFBP-3 has been shown to improve muscle protein synthesis in burn patients (Herndon et al., 1999). Follow-up *in vitro* experiments should examine other signaling pathways that are known to be influenced by levels of circulating cytokines/hormones that are dysregulated in burn serum.

In conclusion, exposure of healthy muscle cells to media containing burn serum results in impaired myogenesis and a reduction in myotube size, implicating humoral factors as a contributor to burn-induced long-term muscle loss. While the reduction in myotube size appears to be via blunted p70S6k signaling and reduced protein synthesis, it is not clear exactly which factors in burn serum are contributing to this. Future studies should consider proteomic techniques to fully characterize the components altered in burn serum that may regulate burn-induced atrophy and/or impaired muscle regrowth. A greater understanding of which specific factors in burn serum contribute to reduced myogenesis and muscle cell size will be beneficial for developing more targeted therapies to counteract burn-induced skeletal muscle loss.

## Author contributions

Conception and design of the experiments: KC, MS, EM, and MB. Collection, analysis, and interpretation of data: KC, MS, EM, SW, ST, JC, and MB. Drafting the article or revising it critically for important intellectual content: KC, MS, EM, and MB.

### Conflict of interest statement

The authors declare that the research was conducted in the absence of any commercial or financial relationships that could be construed as a potential conflict of interest.
